# Novel mutation in NLRP3 Exon 7 results in sensorineural hearing loss without chronic inflammation

**DOI:** 10.1186/1546-0096-13-S1-O37

**Published:** 2015-09-28

**Authors:** L Broderick, S Cherukumilli Grevich, C Putnam, H Hoffman

**Affiliations:** 1 University of California, San Diego, La Jolla, USA; 2 Rady Children's Hospital, San Diego, San Diego, USA; 3 University of Washington, Seattle, USA; 4 Ludwig Institute of Cancer Research, La Jolla, USA

## Introduction

Cryopyrin-associated periodic syndromes (CAPS) are a spectrum of autoinflammatory disesase with spontaneous activation of the NLRP3 inflammasome, leading to hypersecretion of IL-1b. To date, more than 90 mutations have been described in *NLRP3,*primarily in exon 3, leading to the autoinflammatory symptoms observed in CAPS. Here, we describe a family with autosomal dominantly inherited, unilateral sensorineural hearing loss, found to have a novel mutation in exon 7 of *NLRP3*.

## Objective

To evaluate the clinical and genetic features of a 2-generation pedigree with unilateral sensorineural hearing loss.

## Methods

Patient data, including audiologic studies, and detailed family history was obtained. DNA was isolated using saliva samples from all family members and Sanger sequencing of all 9 exons of *NLRP3* was performed.

## Results

The proband was first identified during an evaluation for recurrent fevers and aphthous stomatitis, occurring 2-3 times per month, beginning at 4 months of age, without infectious etiology. Episodes were not associated with cold exposures or rash. Physical exam was notable for an otherwise well appearing child, without evidence for inflammation or autoantibodies. At the age of 5, he was noted to have unilateral sensorineural hearing loss in the range of 4000-8000 Hz, similar to his father. Two younger siblings were noted to have similar symptoms, with similar age of onset, including unilateral hearing loss. Between flares, ESR and high sensitivity CRP were normal in all three children suggesting an absence of chronic inflammation. We identified a novel variant in *NLRP3* exon 7: c.2753 G>A, R918Q in all 4 affected members of this 5-person pedigree. Modeling of the mutation localizes the substituted amino acid residue to the inner surface of the LRR domain and suggests an alteration in charge distribution predicted to affect inter or intraprotein protein-protein interactions.

## Conclusions

While bilateral sensorineural hearing loss has been described in association with Muckle Wells Syndrome and Neonatal onset multi-inflammatory disease (NOMID), both on the CAPS spectrum, in this family, a novel NLRP3 variant was associated with unilateral hearing loss in the absence of serologic evidence for chronic inflammation. This is the first potentially disease associated variant to be described in exon 7 of *NLRP3*.

